# Supplementary material for: Antimicrobial peptides in frog poisons constitute a molecular toxin delivery system against predators
Source: Nat Commun. 2017 Nov 14;8:1495. doi: 10.1038/s41467-017-01710-1 (PMC5686178; doi:10.1038/s41467-017-01710-1)
Supplement: Supplementary file 1 — Supplementary Information [file 41467_2017_1710_MOESM1_ESM.pdf]

Supplementary files

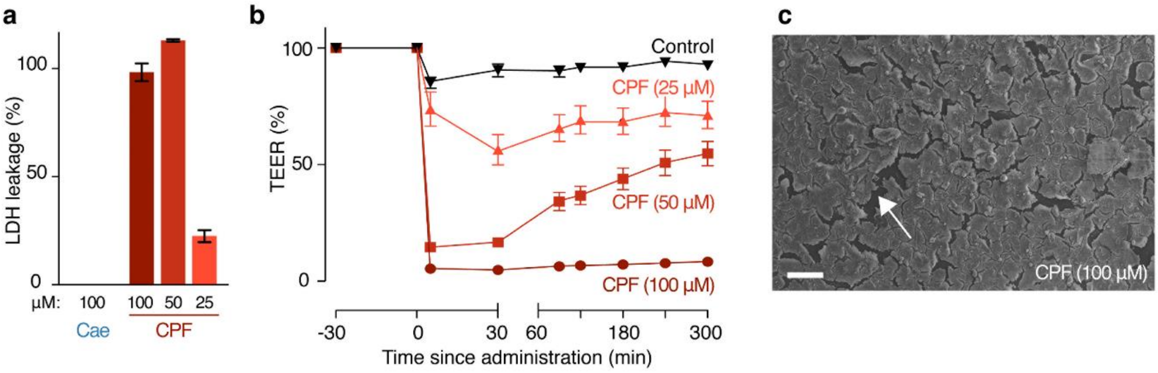

Supplementary Figure 1 | Effect of the AMP caerulein precursor fragment (CPF) on Caco-2 model epithelia. a, Lactate dehydrogenase leakage indicates cell damage in Caco-2 monolayers exposed to CPF but not to the toxin caerulein (Cae) (n = 9; 100 % corresponds to the LDH leakage caused by complete cell lysis as induced by Triton-X). **b,** CPF induces a rapid dose-dependent drop in transepithelial electrical resistance (TEER; n = 7). **c,** Exposure to CPF induces ruptures in Caco-2 monolayers (white arrow), as revealed by scanning electron microscopy. The scale bar represents 100 µm. Data are mean ± s.e.m, error bars not shown when covered by data symbols.

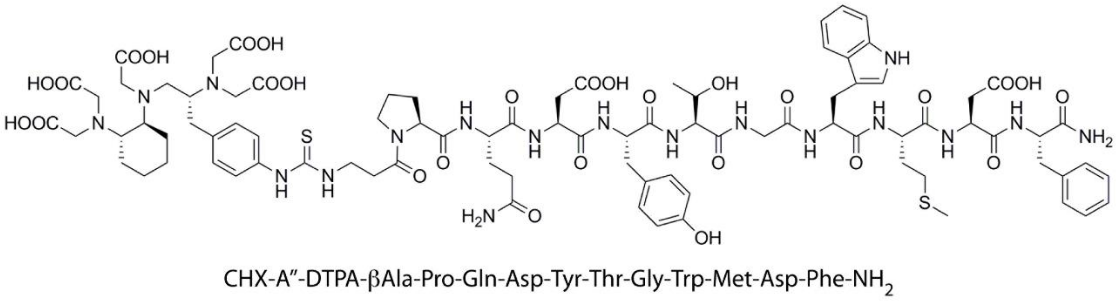

Supplementary Figure 2 | Structural formula of CHX-A''-DTPA-βAla-caerulein used for radiolabeling. To enable radiolabeling, a CHX-A''-DTPA chelator was anchored to the *N*-terminus of caerulein, through use of a β-alanine spacer between chelator and the toxin's sequence. Chemical formula: $C_{87}H_{114}N_{18}O_{28}S_2$, Molecular weight: 1924.0870 Da.

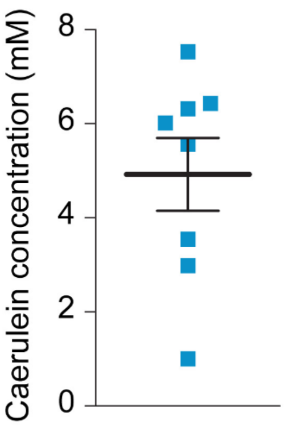

Supplementary Figure 3 | Caerulein concentrations in *Xenopus laevis* skin secretion. Skin secretion of eight individuals was collected and caerulein was quantified in diluted samples using ELISA (See Methods). Data are mean ± s.e.m.

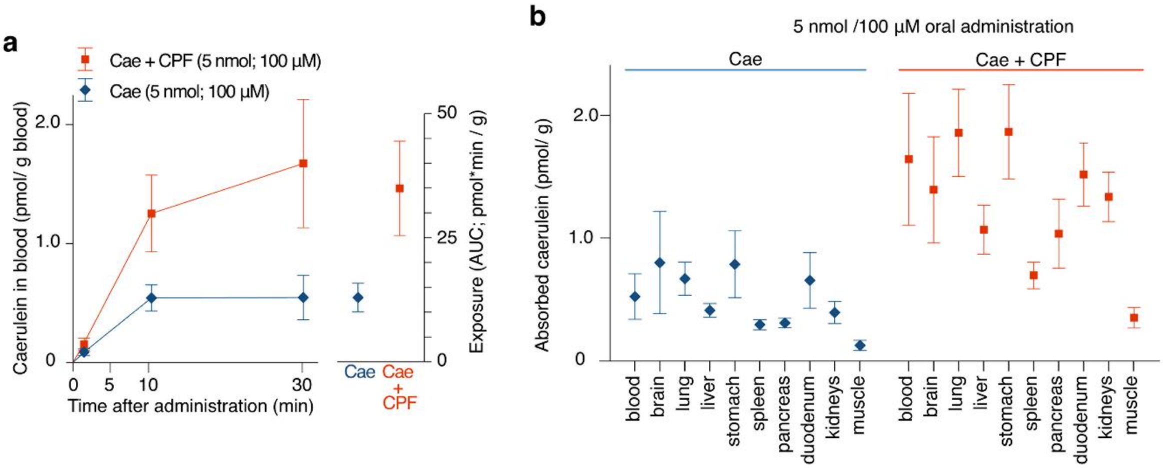

Supplementary Figure 4 | An AMP enhances toxin absorption in a live predator at micromolar concentrations. a, Oral administration of caerulein leads to a more efficient uptake of the toxin into the blood of live snakes when CPF is co-adminstered (left graph, n = 6, linear mixed model, X^2 (1) = 7.9, p = 0.005), resulting in higher systemic exposure as calculated by the area-under-the curve (AUC, right graph). **b,** Co-administration of CPF results in higher caerulein levels in various organs after 30 minutes (n = 6, linear mixed model, X^2 (1) = 10.6, p = 0.0012). Data are mean ± s.e.m. Linear mixed models are explained in the Methods section.

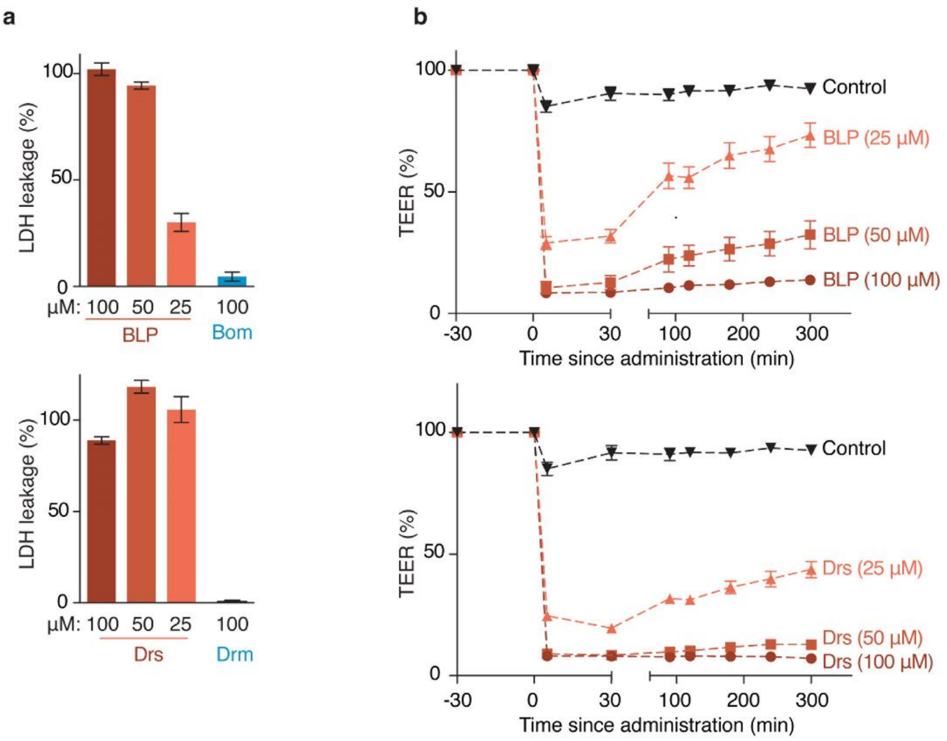

Supplementary Figure 5 | Effect of Dermaseptin-S1 (Drs) and Bombinin-like peptide-1 (BLP) on Caco-2 model epithelia. a, Lactate dehydrogenase leakage indicates cell damage in Caco-2 monolayers exposed to BLP or Drs but not to the toxins dermorphin (Drm) and bombesin (Bom) (n = 9; 100 % corresponds to the LDH leakage caused by complete cell lysis as induced by Triton-X). **b**, Drs and BLP induce a rapid dose-dependent drop in transepithelial electrical resistance (TEER; n = 6-7). Data are mean ± s.e.m, error bars not shown when covered by data symbols.
